# A Blind Test of the Younger Dryas Impact Hypothesis

**DOI:** 10.1371/journal.pone.0155470

**Published:** 2016-07-08

**Authors:** Vance Holliday, Todd Surovell, Eileen Johnson

**Affiliations:** 1 School of Anthropology & Department of Geosciences, University of Arizona, Tucson, Arizona, United States of America; 2 Department of Anthropology, University of Wyoming, Laramie, Wyoming, United States of America; 3 Museum of Texas Tech University, Lubbock, Texas, United States of America; University of Oxford, UNITED KINGDOM

## Abstract

The Younger Dryas Impact Hypothesis (YDIH) states that North America was devastated by some sort of extraterrestrial event ~12,800 calendar years before present. Two fundamental questions persist in the debate over the YDIH: Can the results of analyses for purported impact indicators be reproduced? And are the indicators unique to the lower YD boundary (YDB), i.e., ~12.8k cal yrs BP? A test reported here presents the results of analyses that address these questions. Two different labs analyzed identical splits of samples collected at, above, and below the ~12.8ka zone at the Lubbock Lake archaeological site (LL) in northwest Texas. Both labs reported similar variation in levels of magnetic micrograins (>300 mg/kg >12.8ka and <11.5ka, but <150 mg/kg 12.8ka to 11.5ka). Analysis for magnetic microspheres in one split, reported elsewhere, produced very low to nonexistent levels throughout the section. In the other split, reported here, the levels of magnetic microspherules and nanodiamonds are low or nonexistent at, below, and above the YDB with the notable exception of a sample <11,500 cal years old. In that sample the claimed impact proxies were recovered at abundances two to four orders of magnitude above that from the other samples. Reproducibility of at least some analyses are problematic. In particular, no standard criteria exist for identification of magnetic spheres. Moreover, the purported impact proxies are not unique to the YDB.

## Introduction

The Younger Dryas Impact Hypothesis (YDIH), first proposed by Firestone et al [1, 2], states that some sort of “major cosmic episode of multiple airbursts/impacts occurred at 12,800 ± 300 calendar years before 1950” [3] and brought on a variety of cataclysms across the Earth’s surface including abrupt climate change, wide spread burning, and extinction of fauna and human groups. This proposed event would have occurred at the Younger Dryas boundary (YDB) at the beginning of the Younger Dryas Chronozone (YDC). The data used to support the YDIH include an array of physical and chemical analyses of samples collected at, above, and below the 12.8ka level at sites across North America and other continents (e.g. [1, 2, 4–8]). These indicators include: magnetic grains with iridium, magnetic microspherules, charcoal, soot, carbon microspherules, glass-like carbon containing nanodiamonds, and fullerenes with ET helium.

The specific nature of the extraterrestrial event has never been clearly identified or explained, and the YDIH itself has gone through a variety of iterations. The dating of the proposed impact is far from clear. Originally placed at 12.9+/-0.10ka BP in early YDIH papers, it was revised to 12.8ka+/-0.15ka by Wittke et al [8] as discussed therein. The standard deviation was doubled by Kennett et al [3] without elaboration although in the title of that paper they use an age range of 12,835–12,735 (12,785 ± 50) cal B.P. with no explanation or discussion. The YDIH also generated considerable scientific skepticism and criticism regarding impact physics, the sorts of indicators that are indicative of impacts, the claimed effects of the purported impact, and the dating of zones with purported impact indicators (e.g. [9–20]). Two fundamental questions persist: Can the results of analyses for impact indicators be reproduced? And are the indicators unique to the YDB, i.e., ~12.8k? This paper presents the results of a test that addresses both questions. Two different labs analyzed identical splits of samples collected at, above, and below the ~12.8ka zone at the Lubbock Lake archaeological site (LL) in northwest Texas. The results between the two labs could not be reproduced, but neither lab produced evidence to support the YDIH at Lubbock Lake. Instead, purported impact indicators were found at a level well over 1000 years younger that the YDB.

Lubbock Lake is located in an entrenched meander of Yellowhouse Draw on the northern outskirts of Lubbock, Texas, on the Southern High Plains. Lubbock Lake is public land, owned by Texas Tech University and governed by the Museum of Texas Tech University. As such, research at Lubbock Lake is subject to the Texas Antiquities Code administered by the Texas Historical Commission. All necessary permits were obtained for the described study, which complied with all relevant regulations.

## Methods

In 2007, J. Kennett, D. Kennett (both co-authors on the original Firestone et al [2] paper and subsequent publications), and VTH met at Lubbock Lake to discuss sampling of the section as a test of the YDIH. All agreed that blind splits of samples would be collected and one set sent to JK, and the other to T. Surovell (University of Wyoming). LL was considered a good candidate for testing the YDIH because the site stratigraphy for the time period of interest is very similar to that at the Blackwater Draw site (aka Blackwater Draw Locality 1 or the Clovis site), 120 km northwest of Lubbock in eastern New Mexico and in the same drainage system as Lubbock Lake [21–25]. Data from Clovis was presented in the original publications in support of the YDIH [1, 2] and continues to be used as a key site in support of the YDIH (e.g. [3, 8]), although the archaeological record has been misstated [13, 19].

At both sites the ~12.8ka zone is at the base of diatomites and diatomaceous lake sediments resting on top of alluvial sands and clays. At the Clovis site the YDB sample section (on the South Bank) is at the base of Unit D diatomite (i.e., “black mat” equivalent), resting on the Unit C brown sand wedge. According to Firestone et al [2] (Supp Data) the YDB is a “layer of fine-grained fluvial or lacustrine sediment that lies at the base of the black mat in the uppermost stratigraphic horizon containing *in situ* mammal bones and Clovis artifacts.”

**Fig 1:**
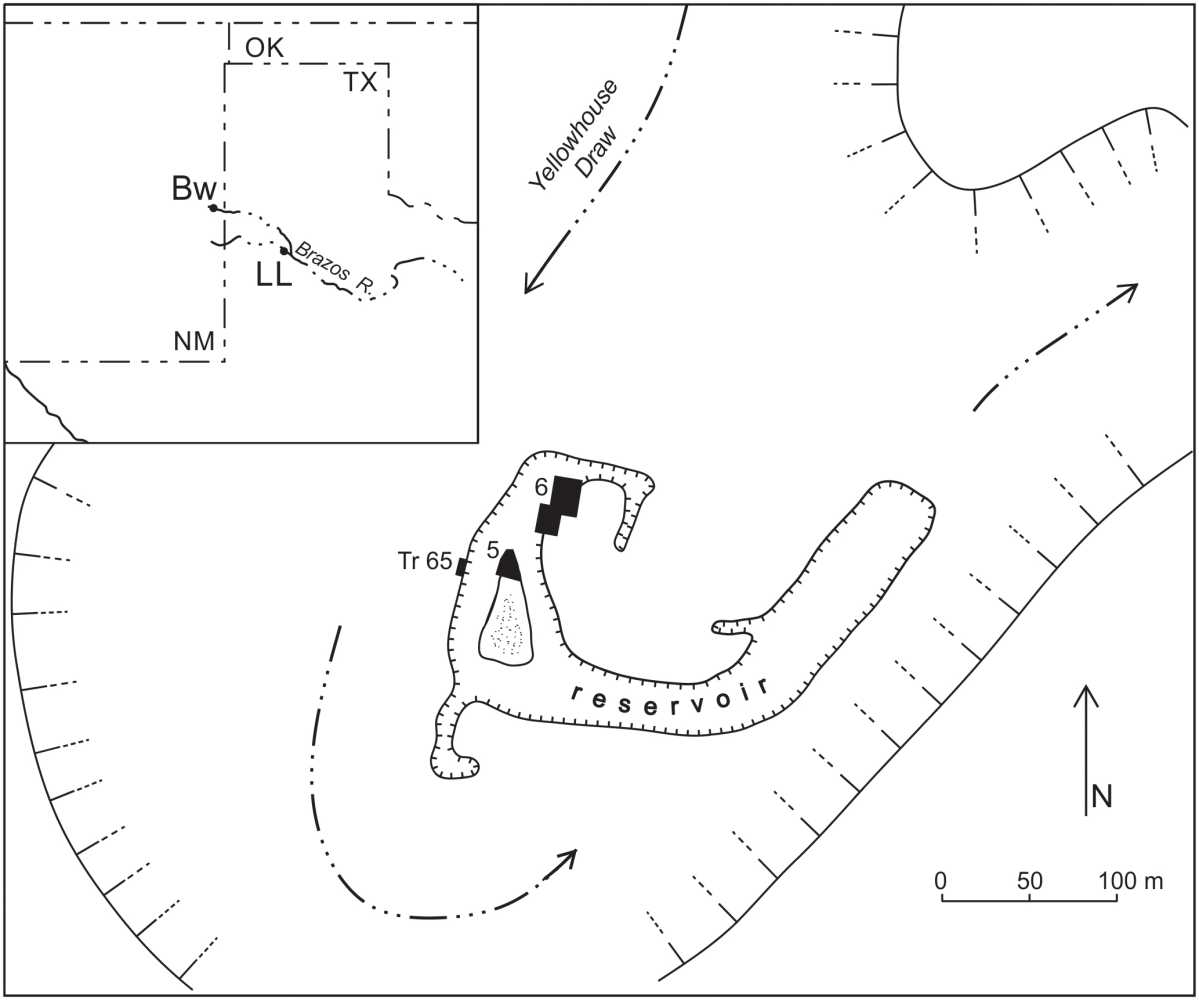
Lubbock Lake in the wide entrenched meander of Yellowhouse Draw. The “reservoir” was excavated in 1936 through valley fill along the inside of the meander. The samples for the blind text were collected from trench 65 (Tr 65) on the outer west wall of the reservoir. Excavation areas 5 and 6 produced lithostratigraphic, biostratigraphic (megafauna), radiocarbon, and archaeological data correlateable to trench 65. Inset shows Lubbock Lake (LL) along Yellowhouse Draw just above its confluence with Blackwater Draw, both tributaries to the Brazos River. The location of the Blackwater Draw site (Bw), also known at the Clovis site, is shown in the upper reaches of the draw.

**Fig 2:**
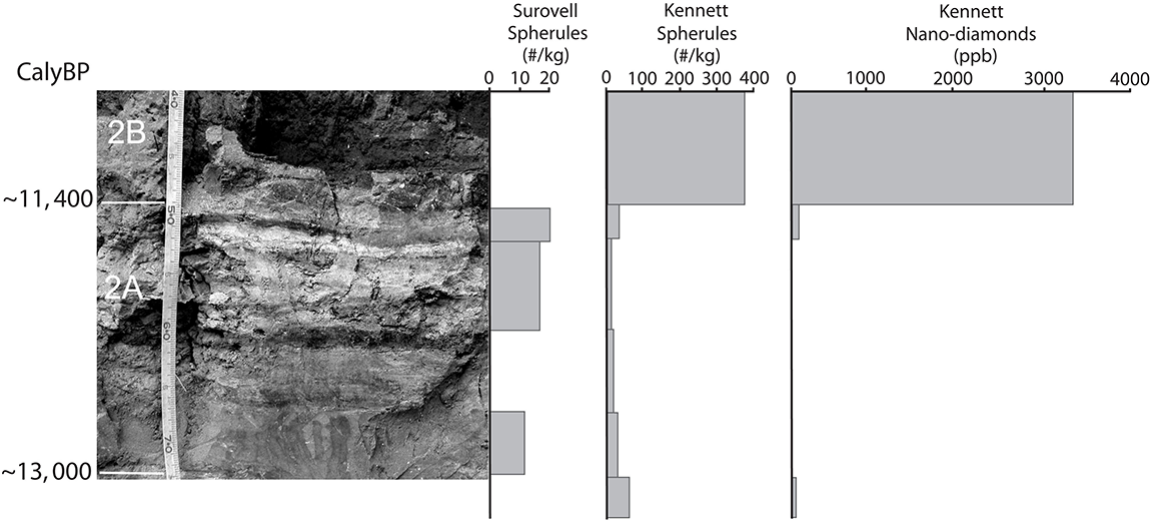
At left is the microstratigraphy of stratum 2 exposed in trench 65 when the sampling for the blind test was underway. Radiocarbon age control is at far left. The scale is in centimeters and millimeters and corresponds to the sampling intervals in Table 1. Right of the image are the results of analyses for magnetic microspheres and nanodiamonds (from [11, 28] see also S1 Table].

The sample section at Lubbock Lake (trench 65) is on the west side of an old, defunct reservoir excavated in the floor of Yellowhouse Draw in 1936 and exposing the valley fill (Fig 1) [24–26]. That fill contains a geological and archaeological record spanning the past ~13,000 calendar years. In trench 65, the basal alluvium (stratum 1) is a fining upward sequence of gravel to sand to clay (Table 1). In general, the contact with the overlying lake beds (stratum 2A) is abrupt, very clear, and horizontal [25–27] including trench 65 (e.g., figure 4 in [26]; 2–3 meters south of—left of—the 2007 sample section in Fig 2). But in the sampled section of the trench, the upper clays grade up into 2A over a thickness of 6cm (67-73cm depth) (Table 1; Fig 2). The classic expression of stratum 2A in the sample section consists of ~18cm (49-67cm depth in Table 1) of interbedded white to light gray laminated diatomite separated by black to dark gray lenses of mud. Above 2A is stratum 2B, resting conformably on 2A and generally comprised of homogeneous gray mud. Locally, 2B, especially lower 2B, is dark gray to black and weakly to moderately bedded.

**Table 1:** Sample context and field descriptions of sampled section at Lubbock Lake.

Depthcm*	Random sample number	Stratum	Description
40–49	3	2B	dark gray mud, weakly bedded
49–53	1	2A	laminated diatomite with black mud 50-51cm
53–60	5	2A	weakly laminated gray diatomite
60–67	2	2A	weakly laminated gray diatomite; black mud 60-61cm
67–73	4	2A	weakly laminated gray, clayey diatomite; wavy lower boundary; transition from strat 1 to strat 2?
73–75	6	1C	olive gray clay

* depth below top of stratum 2

No radiocarbon samples were collected from either stratum 1 or stratum 2 in the trench 65 section. Dating is based on stratigraphic correlation to dated sections in Area 5, 15–20 m to the east-northeast, and in Area 6, 60–75 m to the northeast (all on the same side of the old reservoir; Fig 1) [24–30]. Dating at Lubbock Lake, as at Clovis, is further supported by: 1) presence of late Pleistocene megafauna (*Mammuthus columbi*, *Bison antiquus*, *Equus mexicanus*, *E*. *francicsi*, *Camelops hesternus*, *Arctodus simus*, *Holmesina septentrionalis*, *Capromeryx*) in the stratum 1 alluvial sands below the lake beds, but only extinct bison and extinct antelope in and above the stratum 2 lake beds [25]; and 2) presence of Folsom artifacts exclusively in the diatomite (and dated ~12,800 to ~12,000 cal years BP at LL and throughout the Great Plains [23, 31–35]), and Plainview, Firstview/Cody, and other unfluted Paleoindian artifacts (dated ~12,000 cal yrs BP and younger at LL and throughout the Great Plains [23, 29, 30, 33–35]) in the muds immediately above the diatomite. If indicators used to argue for the YDIH are present at LL, they should be at the base of stratum 2A, the stratigraphic and geochronologic equivalent of the base of stratum D at Clovis. The sample most likely to represent the YDB is 67-73cm (sample 4)

Six samples were collected from upper stratum 1, stratum 2A, and overlying 2B in the trench 65 section (Table 1; Fig 2) to test for the reproducibility of lab methods used to extract purported impact indicators and to generally reproduce the stratigraphic sequence at Clovis. The samples were assigned random numbers in the field and sample splits of ~1 kg each were sent to TS and to JK for analysis. Both labs analyzed samples for magnetic grains and magnetic microspherules. JK’s lab also analyzed samples for nanodiamond content. These particles have been considered to be key impact indicators [1–8].

The methods used by TS followed those used for the results published by Firestone et al [2] (as discussed elsewhere in [11, 36]). The methods used by JK followed Firestone et al [2] (as updated in [37, 38]).

## Results and Discussion

The analyses by TS were reported by Surovell et al [11]. The analyses by JK were completed in 2010. An intermediary (W. Alvarez) was selected to compare the stratigraphic and chronologic data provided by VTH with the results generated by JK. That mediator produced a report submitted to JK and VTH (19 February, 2010). The results were never published by JK. The sampling at LL, however, was conducted under a Texas Historical Commission Antiquities Permit 4196. The data, therefore, are public information and were published in a report to the Texas Historical Commission [28]. Those data are discussed below.

**Fig 3:**
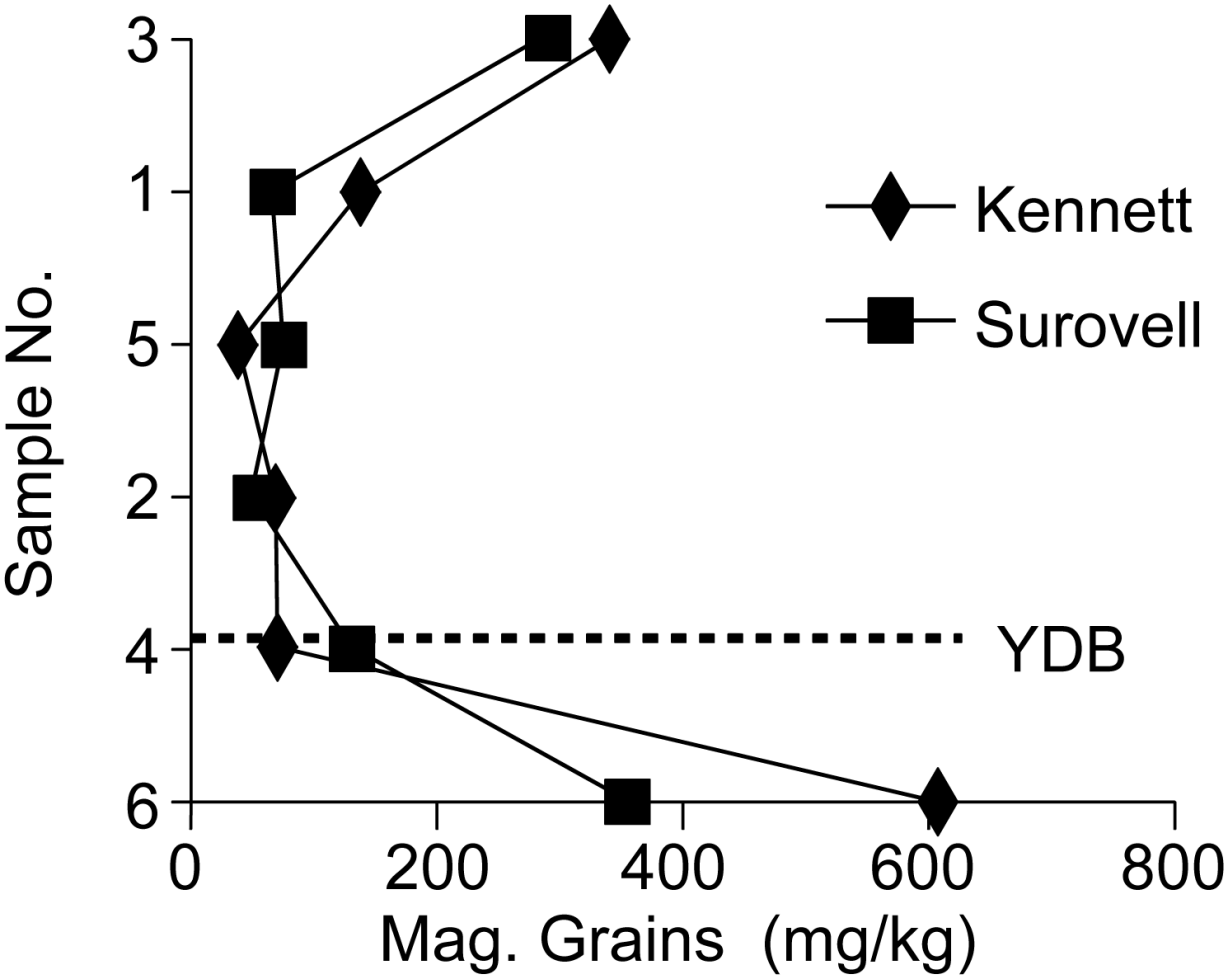
Results of analyses for magnetic grains from Lubbock Lake, stratum 2, trench 65 (from [11, 28]; see also S1 Table).

The data for magnetic spheres generated by Surovell show very low levels throughout the section (Fig 2). Magnetic grains (i.e., all magnetic materials in the sample) show elevated levels at the base of the section (stratum 1) and at the top (stratum 2B), but the overall amounts, all <0.4 gm/kg, are quite low (Fig 3).

The results of the analyses by JK that included recovery of both magnetic spheres and nanodiamonds are striking (Fig 2). The amounts of total magnetic grains recovered by JK are broadly similar to those recovered by TS (Fig 3). Total magnetic grains are most abundant in stratum 1C, at the base of the sample section, and in stratum 2B, at the top of the sampled section. As found by Surovell et al [11], recovery of magnetic spheres by JK was very low with a single outstanding exception. They are much more abundant, by two to four orders of magnitude, in the uppermost sample, from stratum 2B, compared to the other samples (Fig 3). Likewise, nanodiamonds are absent or at very low levels with the exception of the sample from 2B. The uppermost sample yielded nanodiamonds at a level several orders of magnitude larger than any of the other samples (Fig 3). The peak in magnetic spheres and nanodiamonds co-occur and at a stratigraphic level dated ≤11,500 cal yrs BP, at least 1300 years younger than the YDB.

The data from Lubbock Lake clearly raise questions about the meaning of claimed impact indicators, reproducibility of lab methods, and the validity of the YDIH. The methods used by Surovell et al [11] and in particular his inability to find peaks in magnetic spheres were criticized [38]. Surovell [36] enumerated a response. Most generally, LeCompte et al [38] criticized Surovell et al [11] for not following methods that were not published until after 2009. As Surovell [36] (SI 1) pointed out “impact proponents have made *post hoc* modifications to laboratory methods and then criticized prior researchers for not using them.” Nevertheless, he recovered spheres in low abundances, broadly similar to JK except for the uppermost sample. This situation raises a serious question of reproducibility of results: why were spheres recovered by TS but not in zones with abundances purported to be 2–4 orders of magnitude compared to zones below?

**Fig 4:**
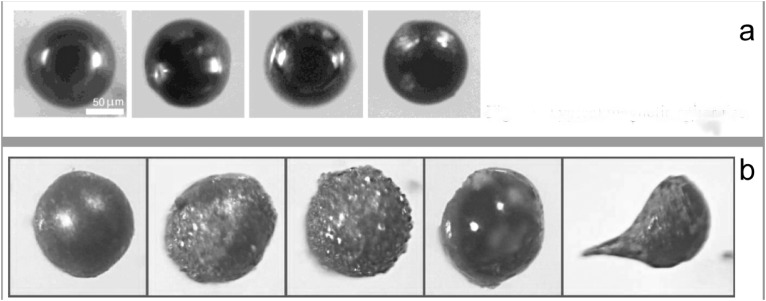
Photomicrographs provided to Todd Surovell by Allen West to aid in spherule identification. A) Fig 13 from an unpublished methodological document titled “Separation of YD Event Markers (8/10/2007).” B) Fig 9 from an unpublished methodological document titled “Younger Dryas Boundary (YDB) Markers” (version 1-1-2010)” (Published with permission).

**Fig 5:**
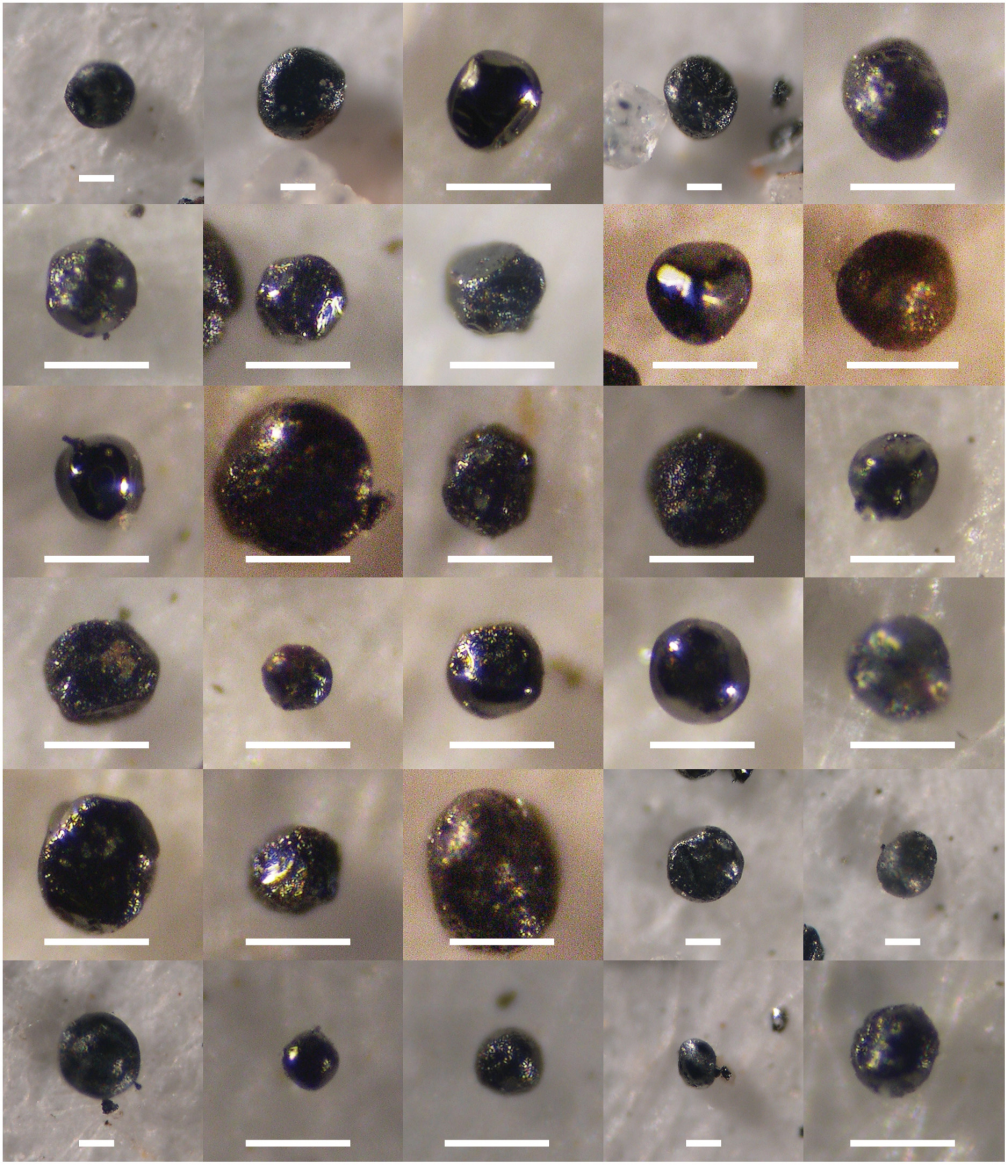
Photomicrographs of magnetic grains from Lubbock Lake sample interval 73–75 cm. How many magnetic spheres do you see? Compare to Fig 4a and to Fig 4b. All scale bars are 50 microns.

That question gets at a more fundamental issue: the identification of magnetic spheres is subjective. In an unpublished methods document titled “Separation of YD Event Markers (8/10/2007)” provided by Allen West, “typical magnetic spherules” were illustrated with the same microspherule images published in Firestone et al [2] (Fig 4a). In January of 2010, after the publication of Surovell’s study, West provided an updated and unpublished version of the protocols titled “Younger Dryas Boundary (YDB) Markers (Version 1-1-2010)” in which “typical magnetic ‘spherules’” were illustrated as having dramatically different morphologies including particles that have rough surfaces and are nonspherical, even including teardrop-shaped sedimentary grains (Fig 4b). Fig 5 illustrates magnetic particles from the LL sample at 73–75 cm. Following the identifications in Fig 4a, the sample in Fig 5 contains very few spheres. But using the illustration in Fig 4b, then almost all of the photomicrographs in Fig 5 contain magnetic spheres. This example clearly shows that sphere counts could vary substantially between samples, even those counted by the same individual. Thus the only major discrepancy between data produced by the Surovell and Kennett labs can be explained by subjectivity in microspherule identification and/or the application of different criteria for microspherule identification.

Firestone [39] and LeCompte et al [38] are also critical of the sampling intervals used in this blind test. However, the original publications on the YDIH [1, 2] provide no specification on sampling intervals. Moreover, most of the key papers in support of the YDIH include samples collected in intervals that vary widely with maximum intervals ranging from 8 to 30cm (Table 2; see also [17]). For example, Table 2 by LeCompte et al [38] show that their sampling intervals at Blackwater Draw varied from 4.5 to 21cm thick. Further, most sampling reported is from in and around the presumed YDB zone rather than throughout thicker or more continuous sections to see if claimed indicators are at other stratigraphic levels.

**Table 2:** YDIH Sampling Intervals by various investigators.

YDIH Study	Sampling Intervals, cm
Firestone et al. [1, 2, 40]	9, 10, 20
Kennett et al. [5]	10
Kurbatov et al. [41]	15
Bunch et al. [7]	10, 15
Israde-Alcantara et al. [37]	10
LeCompte et al. [38]	10, 15, 21
Wittke et al. [8]	8, 10, 15, 30
Wu et al. [42]	10
Bement et al. [43]	10

Species of magnetic microspheres and nanodiamonds were not differentiated. In part, this is because the work by TS was carried out prior to arguments over the origin of various species of microspheres. JK likewise did not perform species identification of either magnetic microspherules or nanodiamonds as part of the most recent study. In any case, the initial arguments that nanodiamonds are clear indicators in support of the YDIH [2, 5] were made before the significance of species identification was fully articulated.

YDIH proponents have argued that some species of magnetic microspheres and nanodiamonds are of terrestrial origin, while others are likely of impact origin [9, 38], but no agreement exists on these issues [12, 17, 43–48]. Bunch et al [7] rejected a criticism of the YDIH noting that the investigators who authored the critique “performed no SEM or EDS analyses to determine whether their spherules are volcanic, cosmic, or impact-related, as stipulated by Firestone et al (2007).” But Firestone et al [2] state (p. 16019) that they were unable to use compositional analyses: “the similarity in composition of YDB magnetic microspherules and magnetic grains (e.g., high Ti) from many sites across North America cannot be explained at this time, but the YDB abundance of microspherules and magnetic grains most likely resulted from the influx of ejecta from an unidentified, unusually Ti-rich, terrestrial source region and/or from a new and unknown type of impactor.”

The greater point here is that according to the Kennett lab, in a study expressly designed to be a test of the YDIH, both magnetic microspherules and nanodiamonds were found to co-occur at significantly elevated levels in a discrete stratigraphic context at LL but not at the YDB onset. Kennett et al [5] state that nanodiamonds “were not detected above or below the YDB layer at any site tested” among the three that they studied. Subsequently, Kennett et al [3] argue that “In a number of sedimentary sections, individual types of YDB-like proxies have been observed intermittently in relatively low abundances outside of the YDB layer. However, only the YDB layer exhibits distinct abundance peaks in multiple impact-related proxies and, as such, forms a distinct, widely distributed event horizon or datum layer, similar, for example, to a geochemically distinctive volcanic tephra layer and the iridium rich K—Pg impact layer.” This statement is another post-hoc argument. Many of the key papers on the YDIH, including the first widely disseminated journal article [2], describe and discuss a YDB layer identified on the basis of just one or a few claimed impact proxies (e.g., carbon spheres, magnetic spheres or nanodiamonds [4–6, 8, 38, 41]).

Data from a number of sites show that claimed YDB indicators are not unique to the YDB zone [4, 5, 8, 11, 16]. Some studies illustrate multiple peaks in claimed YDB indicators. Firestone et al [2] document: double carbon spherule and double charcoal peaks at Chobot; the magnetic grain and spherule peak higher than the main carbon spherule peak at Chobot; two Iridium peaks and one carbon spherule peak matching neither Ir peak at Lake Hind; and a variety of peaks that do not match up at Topper. Multiple peaks in claimed YDB indicators also are illustrated by Israde-Alcantara et al [37], and Bunch et al [7].

In their listing of sites with evidence in support of the YDIH, Kennett et al [3] note that nine other sites are poorly dated or undated but “the stratigraphic context of a proxy-rich layer or samples at these sites supports a YDB age.” These sites are Chobot, Alberta; Gainey, MI; Kangerlussuaq, Greenland; Kimbel Bay, NC; Morley, Alberta; Mt. Viso, France/Italy; Newtonville, NJ; Paw Cove, MD; and Watcombe Bottom, United Kingdom.” But this interpretation represents circular reasoning, i.e., these sites yielded supposed impact indicators, therefore they must be of YDB age. However, these sites also could be evidence for multiple “impact proxies” at other times. Indeed, the carbon sphere “impact indicators” at Gainey [2] were dated as late Holocene and modern [15, 39], and late Holocene at Chobot [39]. At Newtonville, late Wisconsin-age sediment yielded more magnetic microspherules than the upper, younger loamy sand of claimed YDB age [42].

Radiocarbon dating at other localities used to support the YDIH [3, 8] also suggests that claimed YDB indicators are not unique to the YDB. At Barber Creek, the claimed YDB zone is at ~100cm below the surface, but a zone with in situ wood charcoal dated to 10,500±50 radiocarbon yrs BP (~12,477 +/-38k cal yrs BP) is documented below 100cm [49]. The large standard deviation for the modeled age of the YDB at Barber Creek, 12,865+/-535 cal yrs BP [3], easily accommodates the high precision date on the charcoal from below the spherule zone. For Bull Creek, Oklahoma, the investigators [43] who participated in all of the field sampling identified the sampling zone for the impact proxies as 307-312cm depth. The radiocarbon sample of ~11,070+/-60 radiocarbon yrs BP (12,935+/-86 cal yrs BP) years is from 298-307cm. It is a bulk sample on organic matter from a soil A horizon, thus representing a mean residence time for the soil carbon. The purported impact proxies at Bull Creek are older than ~12,935 cal yrs BP by some unknown age and also are found in abundance in strata <3000 yrs old. Contrary to Kennett et al [50], these issues of purported impact indicators appearing in sections older and younger than the YDB have never been addressed by YDIH proponents.

Haynes et al [51, 52] and Pigati et al [16] were able to extract some indirect indicators from YDB zones at a variety of sites, but their work also extracted high levels of these “indicators” from samples ranging in age from 40ka to modern. Firestone et al [53], LeCompte et al [38, 54], and Wittke et al [8] accept the methods and some results of that work, but elsewhere Bunch et al [7] (including Kennett and Firestone as co-authors) reject or do not address the contradictory data presented by Pigati et al and Haynes et al. No explanation is provided as to why: 1) the data are accepted if they support the YDIH; but 2) the data in the same publications are rejected if they suggest impacts at other times or other mechanisms for producing the “indicators.”

Siliceous scoria from Abu Hureyra, Syria, is offered as evidence for high temperature melting uniquely associated with the YDB [7, 8]. Thy et al [55], however, reproduced the scoria with lower temperature fires and attributed the particles to anthropogenic burning. They also found siliceous scoria from multiple levels at Abu Hureyra and other sites in the region “dated approximately to between 10,200 and 13,200 years ago and thus span about 3000 years. We found no evidence to suggest that they concentrated at the beginning of the Younger Dryas (~12,900 years ago)” [55].

One striking correlation in this study is that the high levels of magnetic spheres and nanodiamonds at Lubbock Lake are in the darkest deposits, i.e., those with the higher levels of organic carbon (OC). Measured levels of OC are low [27], but that trend (dark gray to black color but low OC) is common for organic-rich soils and sediments buried for even a few hundred years [56]. The association of claimed impact proxies with organic-rich sediment and soils, independent of their age, has been noted since the initial publications by Firestone et al [1, 2] [4, 7, 16, 36, 43, 50, 57]. According to investigations by YDIH proponents, the YDB zone is associated with a “black mat” (i.e., soils or sediments high in organic matter [58, 59]) at 15 of 29 key localities [19]. This association led Pigati et al [16] and Holliday et al [19] to suggest a link between accumulation of impact “proxies” and soils and wetland deposits.

## Conclusions

The results of analyses of blind samples collected at the Lubbock Lake site to test the YD impact hypothesis produced no evidence of an extraterrestrial impact at the YDB. The results from one lab show no peak in magnetic grains nor in magnetic microspheres but data from another lab shows significantly elevated levels of purported impact indicators (magnetic microspherules and nanodiamonds) at ≤11,500 cal yrs BP, well over 1000 years later than the YDB. These results are consistent with a growing body of data that shows that claimed impact indicators are found in deposits both older and younger than the YDB.

The expanded age range for the YDB is placed at 12,800 +/- 300 cal yrs BP by Kennett et al [3] (indicated as a range of 100 years in the title of that paper), but modeled age ranges with standard deviations of >300 years up to 2405 years are presented for layers of claimed impact indicators at nine sites [3]. These layers are argued to represent the YDB based on the premise that if they could be YDB age (the large modeled standard deviations overlap with the 600 interval now proposed for the YDB) they therefore must represent the YDB. But these layers with claimed impact indicators could just as well or perhaps more likely date to some other time. In addition to Lubbock Lake, other sites discussed above (e.g., Bull Creek, Barber Creek, Blackville, Chobot, Gainey, Newtonville) yielded claimed impact indicators that are not or likely not YDB age. Does this mean that there were multiple impacts in the late Pleistocene and Holocene that have gone unnoticed in the geologic record? Or can other origins account for the materials whose origins have been argued to be solely from extraterrestrial impacts?

For the questions presented at the outset of this paper, several answers can be provided. The identification of magnetic spheres is subjective and, therefore, their use as a proxy for an extraterrestrial event is meaningless until criteria are agreed upon for their identification. More generally, purported indicators in support of the YDIH are clearly not unique to the YDB.

To move forward and better understand what happened at the YDB and also to understand the meaning of purported impact markers, stratigraphic sections with continuous records of sedimentation through the late Pleistocene and Holocene must be sampled throughout at close intervals and dated using high precision methods. To date, only Bement et al [43] report such an approach and their data show peaks in possible impact indicators above and below the YDB. Further, agreed upon criteria must be established for microspherule identification; otherwise counts of spheres are pointless. Complete stratigraphic descriptions of sampled sections also are needed, indicating sediment lithologies, weathering zones, soil morphology, and erosional unconformities. Few are available from among the dozens of sites with claimed impact proxies but are critical for evaluating the depositional context of impact proxies and the interpretation of numerical dates.

## Supporting Information

S1 Table Data from the blind splits collected at Lubbock Lake, strata 1C, 2A, 2B. (DOCX)
