# Supplementary material for: A Blind Test of the Younger Dryas Impact Hypothesis
Source: PLoS One. 2016 Jul 8;11(7):e0155470. doi: 10.1371/journal.pone.0155470 (PMC4938604; doi:10.1371/journal.pone.0155470)
Supplement: S1 Table — (DOCX) [file pone.0155470.s001.docx]

S1 Table. Data from the blind splits collected at Lubbock Lake, strata 1C, 2A, 2B.

| **Sample number** | **Depth**  **cm** | **Surovell (*11*)** | | **Kennett (*38*)** | | |
| --- | --- | --- | --- | --- | --- | --- |
|  |  | **Magnetic fraction**  **(mg/kg)** | **Magnetic Spherules**  **(#/kg)** | **Magnetic fraction**  **(mg/kg)** | **Magnetic Spherules**  **(#/kg)** | **Nanodiamonds**  **(ppb)** |
| 1 | 40-49 | 285 | 0 | 134.5 | 31 | 45.0 |
| 2 | 49-53 | 64 | 20 | 69.4 | 18 | 0.4 |
| 3 | 53-60 | 75 | 17 | 333.6 | 381 | 3360.0 |
| 4 | 60-67 | 53 | 0 | 70.4 | 26 | 0.6 |
| 5 | 67-73 | 132 | 13 | 39.5 | 13 | 0.8 |
| 6 | 73-75 | 357 | 0 | 601.1 | 40 | 24.0 |
